# Casimir interaction driven by hyperbolic polaritons

**DOI:** 10.1515/nanoph-2024-0065

**Published:** 2024-05-09

**Authors:** Yang Hu, Xiaohu Wu, Haotuo Liu, Xiuquan Huang

**Affiliations:** School of Power and Energy, Northwestern Polytechnical University, Xi’an 710072, Shaanxi, P.R. China; Shandong Institute of Advanced Technology, Jinan 250100, Shandong, P.R. China; 12433 Key Laboratory of Advanced Manufacturing and Intelligent Technology, Ministry of Education, Harbin University of Science and Technology, Harbin 150080, P.R. China

**Keywords:** Casimir interaction, natural hyperbolic material, v-HPs, s-HPs

## Abstract

Casimir interaction is an intriguing phenomenon that is induced by electromagnetic quantum fluctuations, which dominates the interaction between microstructures at small separations and is essential for micro- and nano-electromechanical systems (MEMS and NEMS). However, Casimir interaction driven by hyperbolic polaritons remains an unexplored frontier. In this work, we investigate the Casimir interaction between natural hyperbolic material hexagonal boron nitride from the perspective of force distribution with different optical axis orientations for the first time. The attractive Casimir force is remarkably enhanced due to the excitation of volume-confined hyperbolic polaritons (HPs). Furthermore, distinct repulsive contributions to the force are observed due to surface-confined HPs that only exist when the optical axis is in-plane. The HPs are associated with a striking thickness dependence of spectral force properties, suggesting that the discrete volume-confined HPs lead to the attractive-repulsive transition of Casimir force. This work sheds light on the relation between HPs and the vacuum fluctuation-induced force, which could offer new opportunities for the development of the MEMS and NEMS.

## 1 Introduction

When two neutral parallel slabs are very close to each other, a force arises from quantum fluctuations of electromagnetic fields [1]–[4], which Casimir first predicted, and considerable progress has been achieved theoretically and experimentally with different configurations in the last few decades [1], [5]–[8]. Casimir interactions have been exploited in many scopes, such as quantum mechanical actuation, quantum levitation or trapping, and micro- and nano-electromechanical systems (MEMS and NEMS) [9]–[12]. It is worth mentioning that the effect of the materials and the geometry of the structure on the force is of fundamental and technical importance, which may lead to the repulsive Casimir force dependence on the orientation of the object [13]–[17].

Hyperbolic material (HM) is a novel material with a special dielectric function tensor that has attracted much interest [18]–[29]. In particular, the principal elements of the dielectric function tensor are different and have opposite signs in the hyperbolic band (HB). Consequently, the isofrequency contour for transverse magnetic (TM) polarization is a hyperboloid. Several works on Casimir forces based on hyperbolic metamaterial were published [30]–[38]. Song et al. show that the Casimir force between hyperbolic metamaterials is significant than that between ordinary dielectrics due to hyperbolic dispersion and investigate the repulsive Casimir force [34], [35]. However, the physical mechanism behind the Casimir interaction between HMs is rarely discussed. It is widely known that the Casimir effect is principally caused by the coupling between the surface plasmons propagating on two metallic mirrors over short distances [39]. As a counterpart, HM can excite a variety of polaritons, such as volume-confined polaritons (v-HPs), ghost hyperbolic polaritons, surface-confined hyperbolic polaritons (s-HPs), shear hyperbolic polaritons, and frustrated modes [19], [26], [40]–[49]. V-HPs and s-HPs with wide wavevector and spectrum excitation properties are essential in the evanescent field. However, the role of HPs in the Casimir force has not been investigated.

In this work, we investigated the Casimir interaction between natural HM. The spectral Casimir forces distribution of bulk hBN, finite-thickness hBN slab, and bulk hBN covered with graphene with different optical axis (OA) orientations are studied. The effects of v-HPs and s-HPs in the type I and II HBs on the Casimir force are explored.

## 2 Modeling and calculation

**Figure 1:**
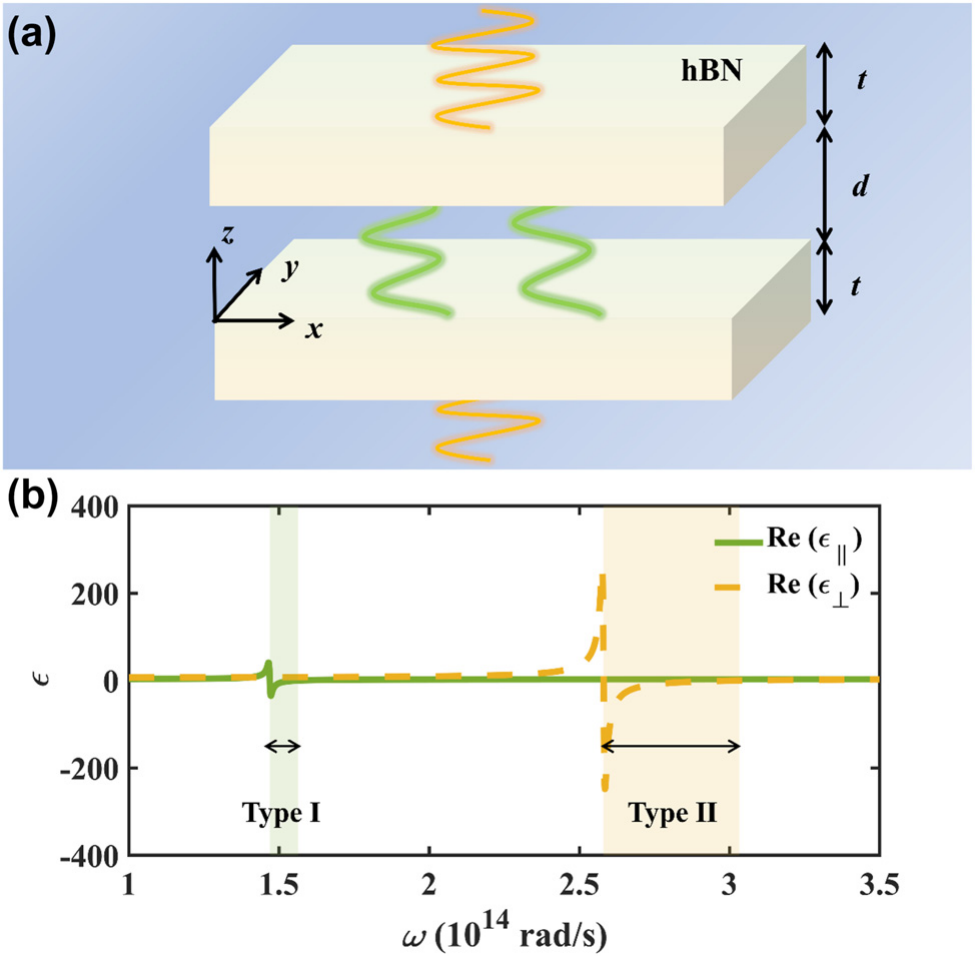
The introduction of the proposed system. (a) The schematic of the Casimir force between hBN slabs. (b) The real part of the dielectric function varying with frequency. The green and orange shaded areas represent the type I and II HBs.

The schematic Casimir interaction between natural HM hexagonal boron nitride (hBN) is shown in Figure 1(a). The hBN slabs are placed in the *x*-*y* plane with thicknesses *t* separated by a vacuum gap of *d*. The dielectric functions of hBN could be described by a single-oscillator Lorentz model, and the detailed parameters are in Refs. [36], [50]:

(1) $$\varepsilon_{m}=\varepsilon_{\infty ,m}\left(1+\frac{\omega_{LO,m}^{2}-\omega_{TO,m}^{2}}{\omega_{TO,m}^{2}-\omega^{2}+i\Gamma_{m}\omega }\right),$$

The hBN is a kind of natural HM, where the small damping coefficients (Γ) compared to the phonon frequencies (*ω*_TO,_ *ω*_LO_), the parallel and perpendicular dielectric functions of hBN have opposite signs in the HBs. The real part of two components of the dielectric function varies with angular frequency, as shown in Figure 1(b). The green shaded area from 1.47 × 10^14^ rad/s to 1.56 × 10^14^ rad/s represents the type I HBs with Re(*ε*_⊥_) > 0 and Re(*ε*_||_) < 0. The orange shaded area from 2.58 × 10^14^ rad/s to 3.03 × 10^14^ rad/s represents the type II HBs with Re(*ε*_⊥_) < 0 and Re(*ε*_||_) > 0.

Following the pioneering Lifshitz theory, the conventional method to calculate the Casimir force is determined by the reflection coefficients of the slabs, which is along the imaginary frequency range of vacuum fluctuations through the Wick rotation. Calculation in imaginary frequency is mathematically convenient, but the physical interpretation of the role of the electromagnetic modes is pretty elusive. On the contrary, Lifshitz’s theory regarding real frequencies and wavevector is applied in calculating the Casimir force per unit area between two hBN slabs [39], [51], [52]:

(2) $$P=\overset{\infty }{\underset{0}{\int }}P\left(k_{x},k_{y},\omega \right)\text{d}k_{x}\text{d}k_{y}\text{d}\omega ,$$

(3) $$P\left(k_{x},k_{y},\omega \right)=\frac{-2\hbar \omega^{3}k}{c^{3}}\mathrm{Tr}\left(Im\left(v\frac{R^{2}e^{-2\omega vd/c}}{I-R^{2}e^{-2\omega vd/c}}\right)\right),$$

where ℏ is the reduced Planck constant, *k* = (*k*_*x*_^2^ + *k*_*y*_^2^)^1/2^ is the wavevector in *x*–*y* space, *v* = (*k* ^2^ − 1)^1/2^, *c* is the light speed in the vacuum, and **I** is the identity matrix. When OA is in-plane, there are depolarization effects, the reflection coefficients *r*_*sp*_ and *r*_*ps*_ are generally nonzero, and the reflection tensor $R=\left(\begin{array}{cc} r_{ss} & r_{sp}\\ r_{ps} & r_{pp} \end{array}\right)$ is no longer diagonal, which could be calculated by the transfer matrix method (TMM) [50]. In the following calculations, the gap distance *d* is fixed to 50 nm.

## 3 Results and discussion

### 3.1 Bulk hBN

The effect of HPs on the Casimir force is analyzed starting from the simplest hBN bulk. When the OA is parallel to the *z*-axis, Casimir forces are attractive over a wide range of HBs and close to zero beyond the HBs. The spectral Casimir forces exhibit two resonant peak-valley pairs located in the type I (very faint) and II HBs, respectively. The two resonances occur at around longitude optical phonons frequencies (*ω*_LO,∥_ = 1.56 × 10^14^ rad/s, *ω*_LO,⊥_ = 3.03 × 10^14^ rad/s) which can be attributed to the epsilon-near-zero (ENZ) modes.

**Figure 2:**
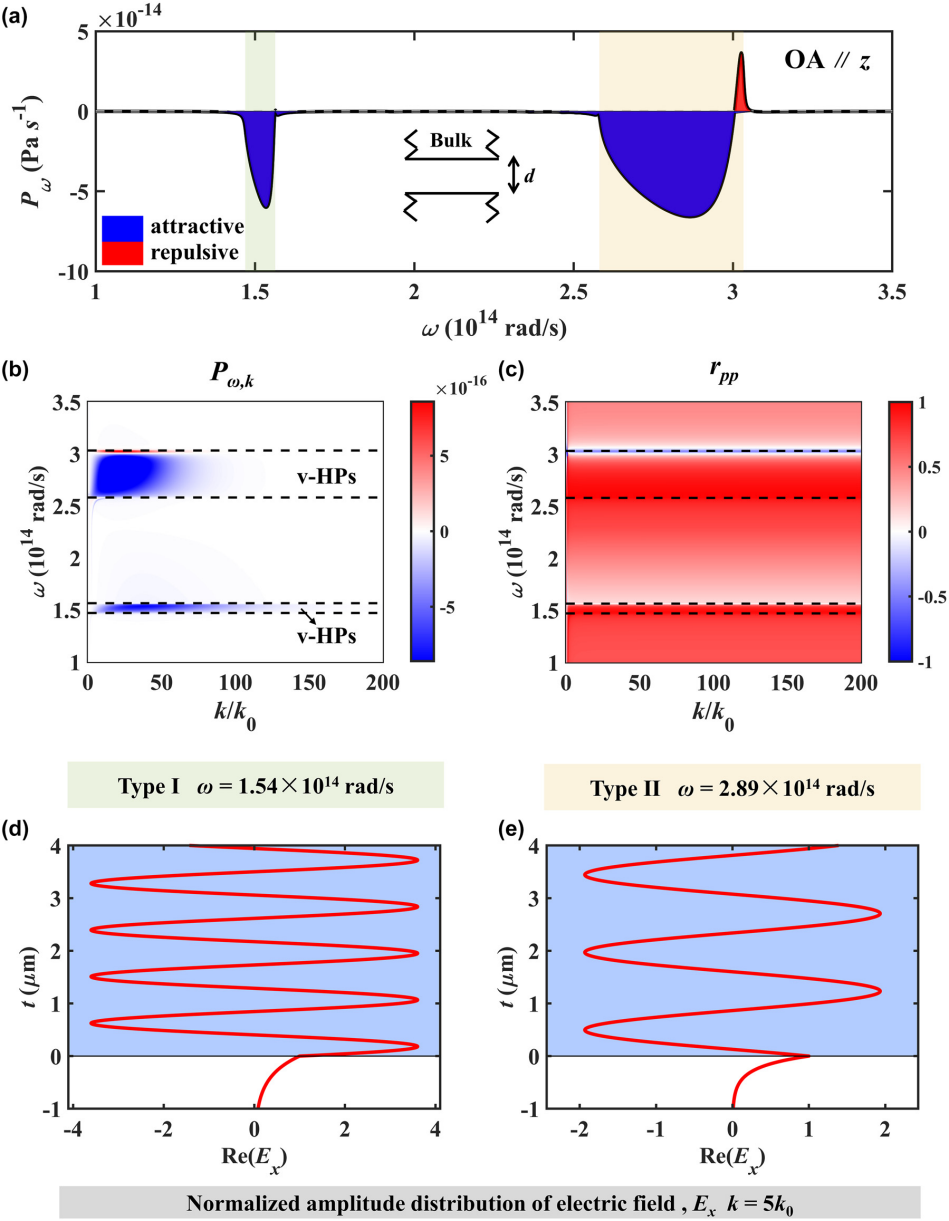
The spectral Casimir interaction hBN bulks when the OA of hBN is parallel *z*-axis. (a) Spectral Casimir force distribution. (b) and (c) Casimir force and reflection coefficients distribution in frequency-wavevector space. (d) and (e) Normalized amplitude distribution of electric field when frequencies are 1.54 × 10^14^ rad/s and 2.89 × 10^14^ rad/s.

To clarify the mechanism of spectrum variation, the Casimir forces distribution in frequency-wavevector space are shown in Figure 2(b). Attractive Casimir force dominates the HBs, while repulsive Casimir force only occurs close to longitude optical phonons frequencies. The Casimir interaction is mainly determined by the component *r*_*pp*_ of the reflection tensor, and underlying physics is shown by the real part of *r*_*pp*_ in Figure 2(c). There is a transition from nearly 1 to −1 in the reflection coefficient as it approaches ENZ frequencies, leading to the emergence of symmetric (lower frequencies) and antisymmetric (higher frequencies) modes, causing the Casimir force to change from attraction to repulsion. To distinguish the types of HPs that contributes to repulsive Casimir forces, the normalized amplitude distributions of the electric fields are shown in Figure 2(d) and (e). In type I and type II HBs, the fields oscillate with the same amplitude from the air/hBN interface (*t* = 0). Therefore, the electromagnetic waves in the HM are propagating waves, which is the characteristic of v-HPs. The Casimir interaction between hBN bulks is driven by v-HPs when OA is out-of-plane.

**Figure 3:**
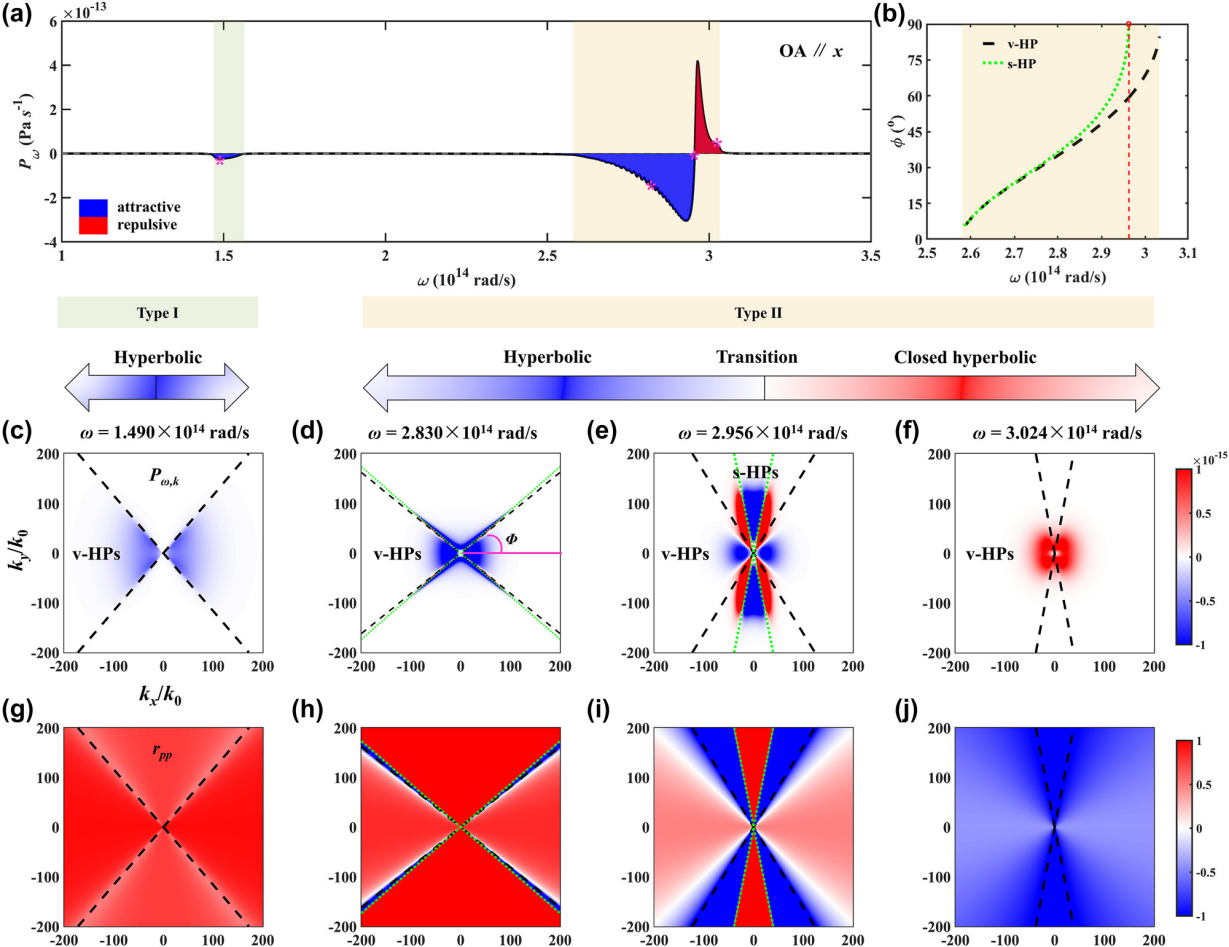
The spectral Casimir interaction hBN bulks when the OA of hBN is along the *x*-axis. (a) Spectral Casimir force distribution. (b) Frequency dependence of the open angle of v-HPs and s-HPs. (c)–(f) The Casimir force distribution in frequency-wavevector space. (g)–(j) Reflection coefficients distribution in frequency-wavevector space. The analyzed frequency points correspond to the magenta asterisks in (a). The black dashed lines are asymptotes of v-HPs, and the green dotted lines are dispersion curves of s-HPs.

The spectral force distribution exhibits a distinctive difference when OA is in-plane and out-of-plane. When OA is in-plane and parallel to the *x*-axis, the Casimir force is attractive in the type I HB, as shown in Figure 3(a). There is only one peak-valley pair at around 2.956 × 10^14^ rad/s in the type II HB and a small peak at ENZ frequency.

The mechanism is investigated by Casimir force distribution in frequency-wavevector space, as shown in Figure 3(d)–(f). When OA is in-plane, the Casimir force exhibits strong anisotropy in wavevector space and varies complicatedly with frequencies. In the type I HB, the attractive Casimir force is distributed in the left and right sides of the wavevector space, as shown in Figure 3(d). The black dashed lines are asymptote lines of v-HPs. When a TM wave is incident on the *x*–*y* plane, the wavevector in uniaxial HM hBN satisfies the relationship (OA is along the *x*-axis):

(4) $$\frac{k_{x}^{2}}{\varepsilon_{⊥}}+\frac{k_{y}^{2}}{\varepsilon_{\|}}+\frac{k_{z}^{2}}{\varepsilon_{\|}}=k_{0}^{2}.$$

The v-HPs could be excited within the range [25], [40]:

(5) $$\frac{\varepsilon_{⊥}k_{y}^{2}+\varepsilon_{\|}k_{x}^{2}}{\varepsilon_{⊥}}<0.$$

The asymptote lines of v-HPs are determined by [25], [40]:

(6) $$\frac{k_{y}}{k_{x}}=\pm \sqrt{-\frac{\varepsilon_{\|}}{\varepsilon_{⊥}}}.$$

The contribution of the electromagnetic model is embodied by the reflection coefficients. The reflection coefficients in the left and right sides of the wavevector space are close to 1, which are much larger than that of other regions, exhibiting hyperbolic dispersion.

In the type II HB, the s-HPs could excite when $1-\varepsilon_{⊥}^{2}<0$ and 1 − *ɛ*_‖_ *ɛ*_⊥_ > 0 meet simultaneously [42]. The green dotted lines are dispersion curves of s-HPs, which could be obtained by [40]:

(7) $$\frac{k_{y}}{k_{x}}=\pm \sqrt{\frac{\varepsilon_{⊥}\varepsilon_{\|}-1}{1-\varepsilon_{⊥}^{2}}}.$$

The open angle (the angle between the OA and the large wavevector branch of the surface/volume mode, shown in Figure 3(d)) of v-HPs and s-HPs are plotted in Figure 3(b). The asymptote lines of v-HPs and the dispersion curve of s-HPs almost overlap at low frequencies and are separated at high frequencies in the type II HB. It is worth noting that v-HPs could excite throughout the type II HB. However, the s-HPs could not excite within *ω* = 2.96 × 10^14^ rad/s ∼ 3.03 × 10^14^ rad/s due to it does not meet $1-\varepsilon_{⊥}^{2}<0$ .

When *ω* = 2.83 × 10^14^ rad/s, the v-HPs could excite in the left and right region, contributing to the attractive Casimir force. S-HPs can be excited in a narrow range at the green dispersion curves with high wavevectors, showing a hyperbolic shape. At this frequency, the excitation positions of v-HPs and s-HPs are very close, which matches the result in Figure 3(b). The reflection coefficients are shown in Figure 3(g), which undergoes a positive-negative transition on both sides of the dispersion curves of s-HPs. When *ω* = 2.956 × 10^14^ rad/s, v-HPs could excite in the left and right sides of the region within the asymptotes (black dashed lines), while s-HP could excite in the up and down the region. Unlike the type I HB, the Casimir force is dominated by the up and down sides of the region. It indicates that the positive-negative transition of the spectral force at this point is caused by the s-HPs. The Casimir force within asymptote lines of v-HPs are attractive, between asymptote lines of v-HPs and dispersion curves of s-HPs are repulsive, and within dispersion curves of s-HPs are attractive. The symmetry mode and antisymmetry mode of s-HPs (both sides of the dispersion curves of s-HPs) cause the attractive-repulsive transition of Casimir force. At the transition point of the spectrum, the open angle of s-HPs is close to 90°. The attraction-repulsion transition of the Casimir force is explained by analyzing the reflection coefficients, as shown in Figure 3(i). The reflection coefficients can be far greater than 1 or far less than −1 with the excitation of s-HPs. When *ω* = 3.024 × 10^14^ rad/s, the Casimir force is repulsive in the whole region and shows a closed hyperbolic shape. The v-HP could be excited in the left and right sides of the region. However, the s-HPs could not be excited because it does not meet 1 − *ɛ*_‖_ *ɛ*_⊥_ > 0.

**Figure 4:**
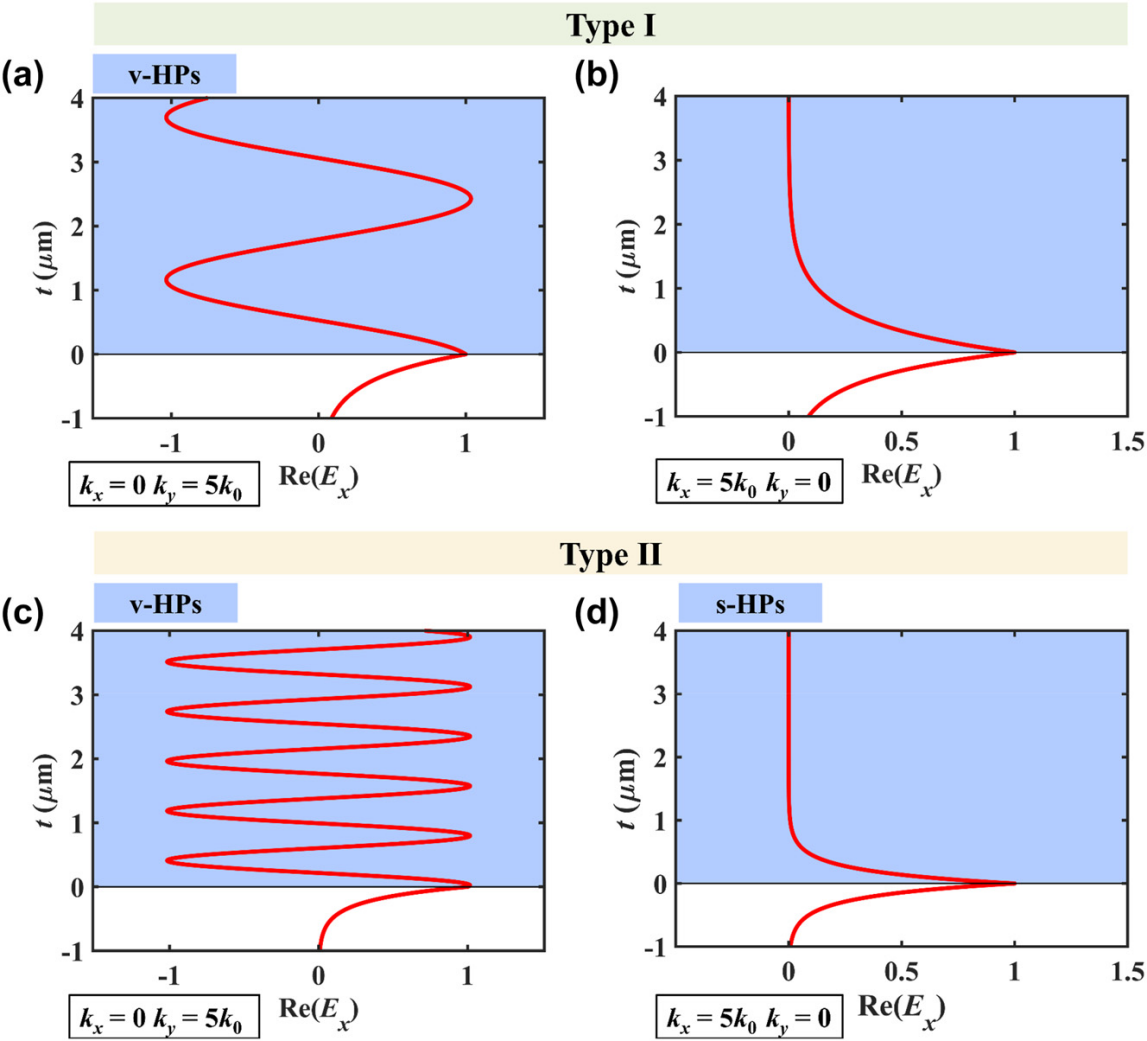
The normalized amplitude distribution of electric field. (a), (b) *ω* = 1.49 × 10^14^ rad/s, (c), (d) *ω* = 2.956 × 10^14^ rad/s.

The electric fields when OA is along the *x*-axis are shown in Figure 4. In the type I HB, the electromagnetic waves in hBN bulks are propagating waves when *k*_*x*_ = 5*k*_0_, *k*_*y*_ = 0. It means v-HPs could be excited and promote Casimir interactions. However, when *k*_*x*_ = 0, *k*_*y*_ = 5*k*_0_, the fields decrease exponentially away from the air/hBN interfaces, indicating the electromagnetic waves are evanescent waves. There is no excitation of HPs in this region. In the type II HB, the electric field for v-HPs (*k*_*x*_ = 5*k*_0_, *k*_*y*_ = 0) are propagating waves, and s-HPs (*k*_*x*_ = 0, *k*_*y*_ = 5*k*_0_) are evanescent waves. The essence of s-HPs is Dyakonov surface waves [53], [54].

### 3.2 Finite-thickness hBN slab

**Figure 5:**
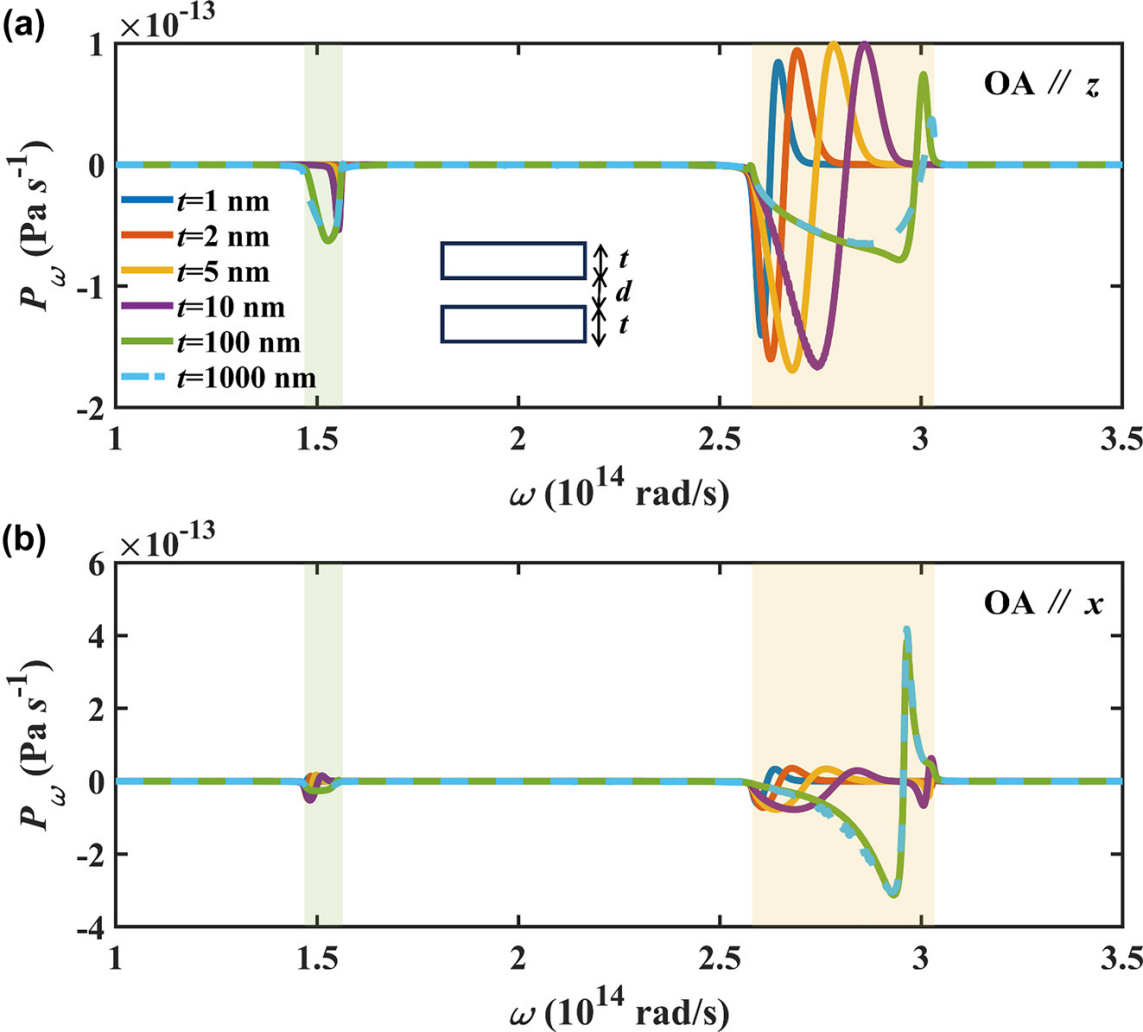
The spectral Casimir force between finite thickness hBN slabs varying with frequency when OA is along (a) the *x*-axis and (b) the *z*-axis.

The spectral Casimir force distribution when hBN slabs at finite thickness will significantly differ from hBN bulk. When OA is out-of-plane, the spectral Casimir force is shown in Figure 5(a). In the type I HB, the Casimir force gradually becomes a narrowband phenomenon with decreasing thickness and approaches the ENZ frequency. The position of the peak-valley pair redshifts with decreasing thickness. When OA is in-plane, finite thickness compared to the bulk is not all attractive in the type I HB, the peak-valley pair will accompany repulsive forces. In the type II HB, there will be two distinct peak-valley pairs at finite thickness. The resonance modes at low frequencies gradually move closer to the transverse optical phonons frequencies with decreasing thickness, while the resonance modes at high frequencies are always at the ENZ frequency with decreasing thickness.

**Figure 6:**
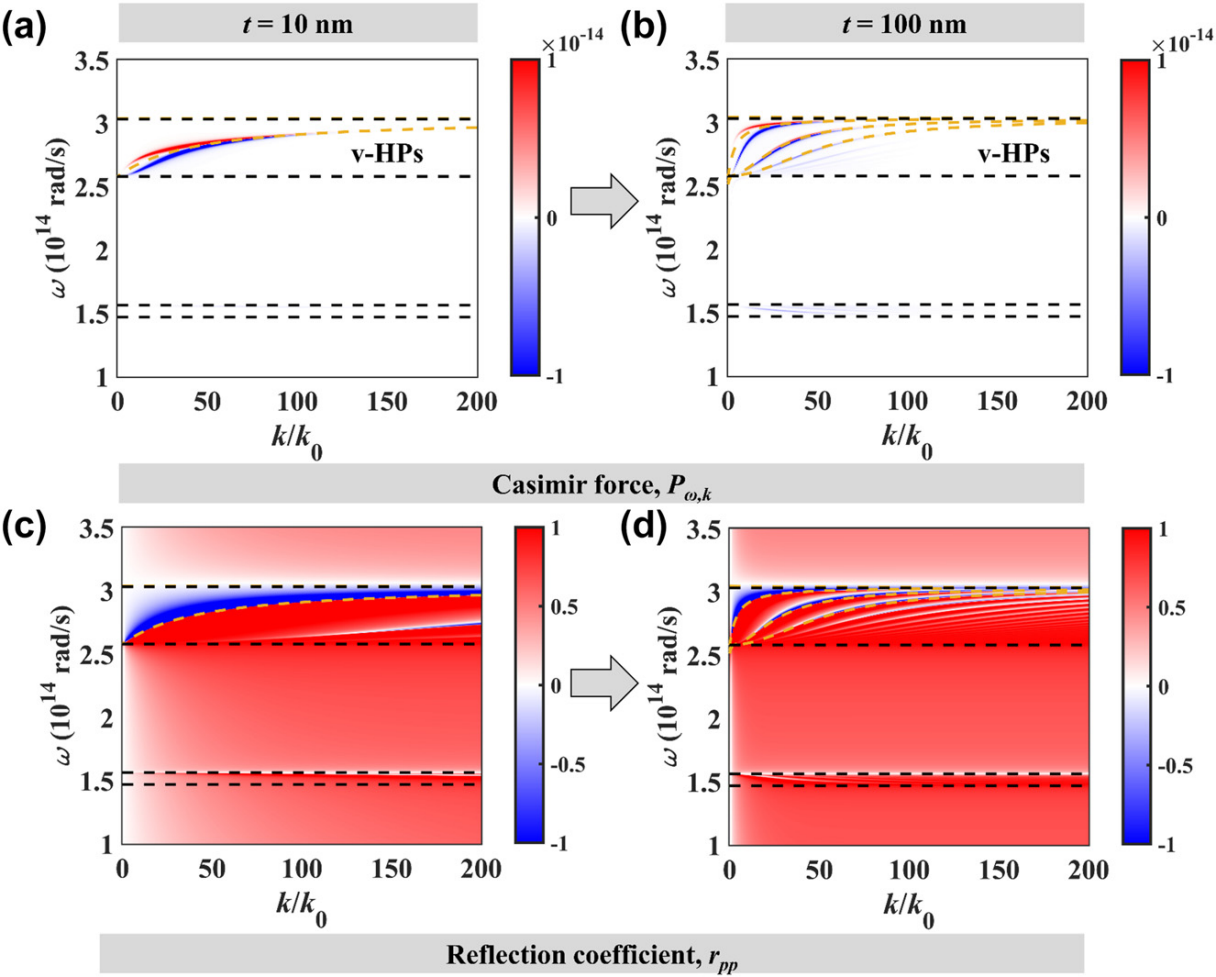
The Casimir force and reflection coefficient distribution in frequency-wavevector space. (a), (b) Casimir force. (c), (d) Reflection coefficient. (a), (c) *t* = 10 nm. (b), (d) *t* = 100 nm.

To investigate the physical mechanisms behind the Casimir force, the distributions of the Casimir force in frequency-wavevector space are shown in Figure 6(a) and (b). When the thickness of the hBN slab is 10 nm, a peak-valley pair splits at small wavevectors and emerges at large wavevectors. The orange dashed lines represent the dispersion lines of v-HPs, which are obtained by [55]:

(8) $$\sqrt{k_{x}^{2}+k_{y}^{2}}=\frac{2\rho }{t}\left[\arctan\left(\frac{\rho }{\varepsilon_{⊥}}\right)+\frac{\pi }{2}l\right],$$

where $\rho =i\sqrt{\varepsilon_{⊥}\left(k_{x}^{2}+k_{y}^{2}\right)/\left(\varepsilon_{⊥}k_{x}^{2}+\varepsilon_{\|}k_{y}^{2}\right)}$ , *l* = 0, 1, 2…. Only 0-order v-HPs could be excited and split into two branches. The splitting phenomenon is similar to the coupled surface polaritons between two aluminum slabs (Ref. [39]) and ultrathin films (Ref. [56]). However, such a splitting does not mean the original modes are always the surface modes. V-HPs are propagating waves approximated with evanescent waves in thin ultrathin films [57]. When the thickness is 100 nm, many orders of v-HPs could be excited. The reflection coefficients for 10 nm and 100 nm are shown in Figure 6(c) and (d). In the vicinity of the v-HPs excitation, there is a positive or negative transition in the real part of reflection coefficients.

**Figure 7:**
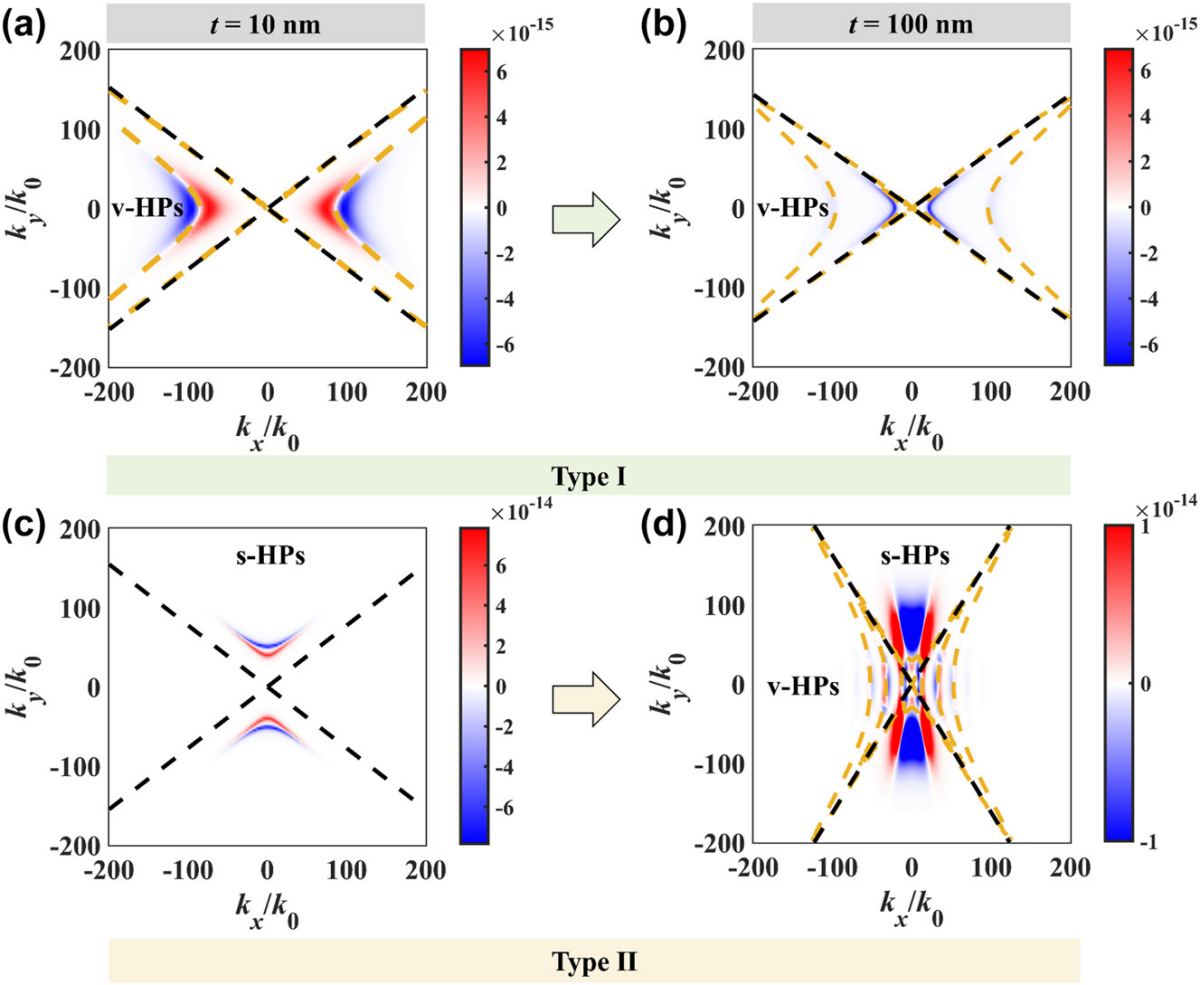
The Casimir force distribution in wavevector space. (a) *t* = 10 nm, *ω* = 1.504 × 10^14^ rad/s, (b) *t* = 100 nm, *ω* = 1.51 × 10^14^ rad/s, (c) *t* = 10 nm, *ω* = 2.82 × 10^14^ rad/s, (d) *t* = 100 nm, *ω* = 2.956 × 10^14^ rad/s.

When OA is in-plane, the Casimir force distributions in wavevector space are shown in Figure 7. In the type I HB, when the thickness is 10 nm, the v-HPs could excite in the left and right, leading to the peak-valley pair in the spectrum. However, when the thickness is 100 nm, the excitation of v-HPs only enhances the attraction and cannot cause repulsive Casimir force between hBN slabs. In the type II HB, when the thickness is 10 nm, the s-HPs could excite in the above and below wavevector space. However, there is no excitation of v-HPs. When the thickness is 100 nm, the s-HPs could be excited in the above and below of wavevector space, and v-HPs could excite in the left and right sides, and exhibit discrete properties.

**Figure 8:**
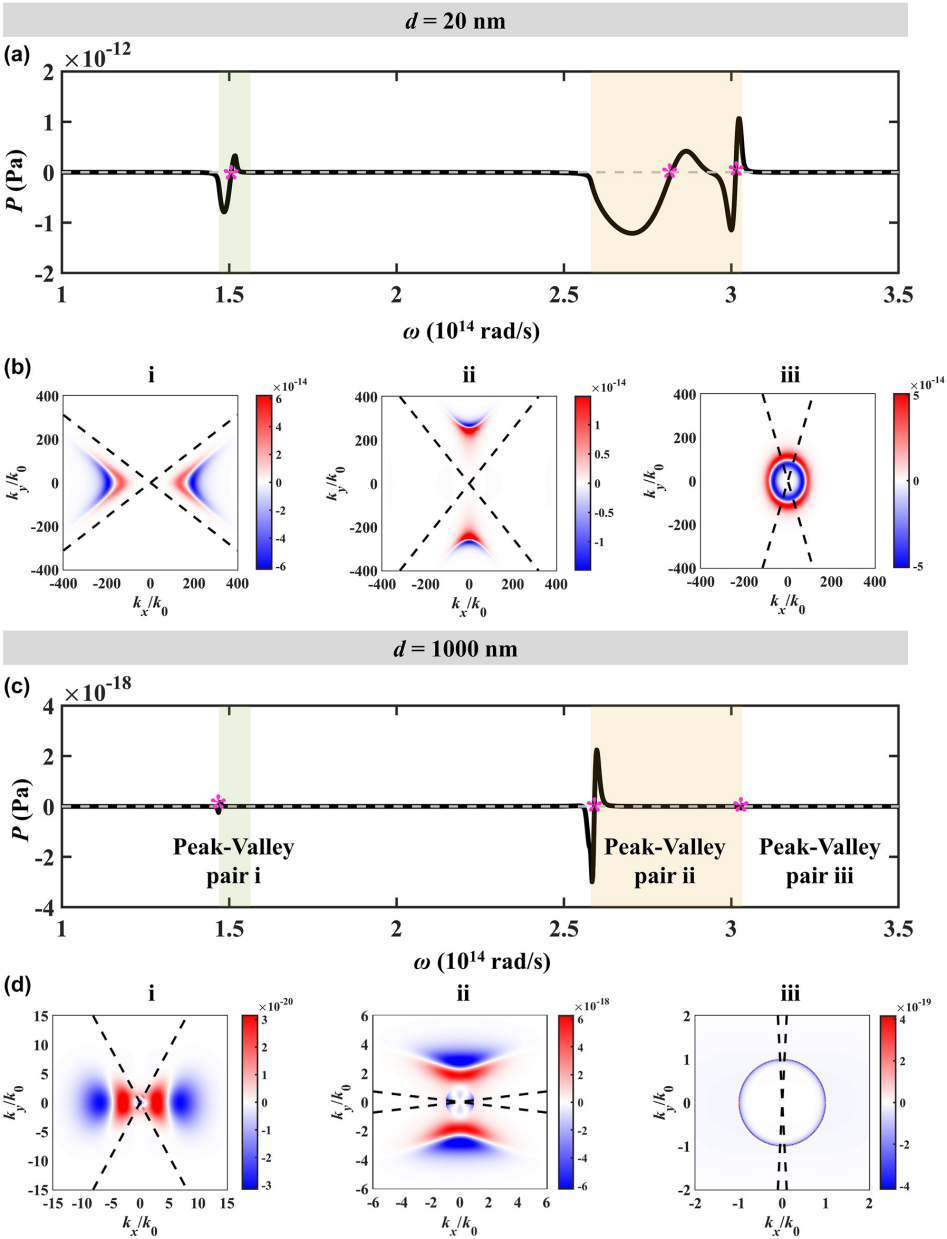
The spectral Casimir force varying with angular frequency (a) *d* = 20 nm and (c) *d* = 1000 nm. The left, middle, and right contours in (b) and (d) are the Casimir force distribution in wavevector space for peak valley pair i, ii, and iii, respectively.

The spectral Casimir forces distribution when the optical axis is along the *x*-axis and the thickness of hBN is 5 nm are shown in Figure 8. There are three peak-valley pairs when the separations are 20 nm and 1000 nm. The spectral Casimir forces when the separation is 1000 nm are much smaller than that of 20 nm due to the range shrinkage of the force in wavevector space. For peak-valley pair i, the Casimir forces are driven by v-HPs, for peak-valley pair ii, the Casimir forces are driven by s-HPs. For peak-valley pair iii, when the separation is 20 nm, the Casimir forces are driven by v-HPs and s-HPs, which is hard to distinguish in ultrathin hBN film. When the separation is 1000 nm, the Casimir force is driven by propagating waves.

**Figure 9:**
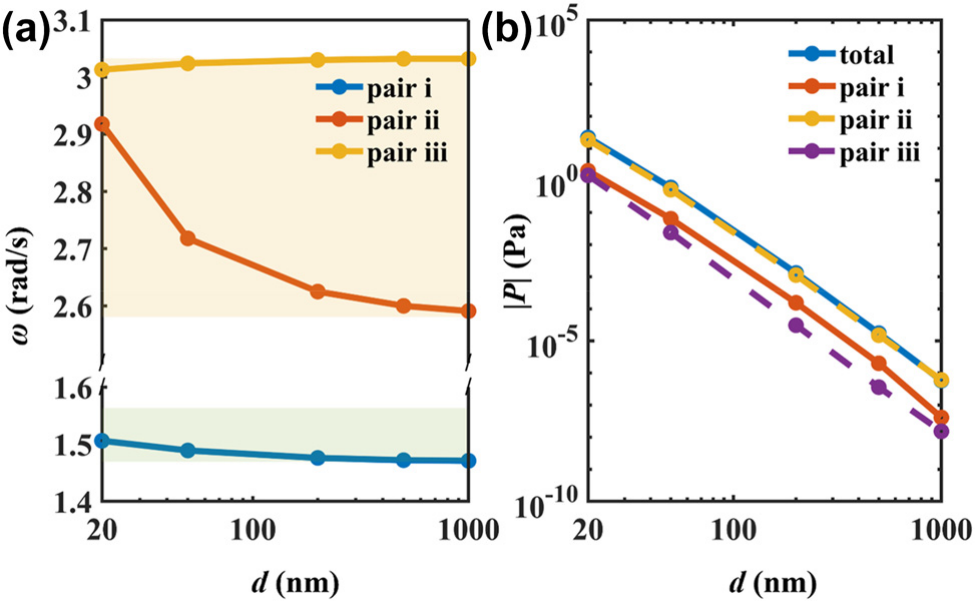
Contributions to the Casimir force between two ultrathin hBN films by the hyperbolic modes. (a) Corresponding frequencies to the force spectral equilibria in the resonant peak-valley pair by the electromagnetic mode. (b) The overall contribution to the force from hyperbolic modes.

The resonant frequencies of three peak-valley pairs varying with separation are shown in Figure 9(a). The peak-valley pair i and iii resonances are persistently pinned around the ENP frequency of type I HB and ENZ frequency of type II HB, almost invariant with the inter-plate separation. However, the peak-valley pair ii resonance frequency decreases with increasing separation, moving closer to the ENP of type II HB. The contributions of three peak-valley pairs on Casimir force varying with separation are shown in Figure 9(b). The Casimir force contributed by these peak-valley pairs decreases exponentially with separation. The peak-valley pair ii very close to the total Casimir force plays a leading role, which indicates that the Casimir forces are driven by the s-HPs for ultrathin hBN films.

### 3.3 Bulk hBN covered with graphene

**Figure 10:**
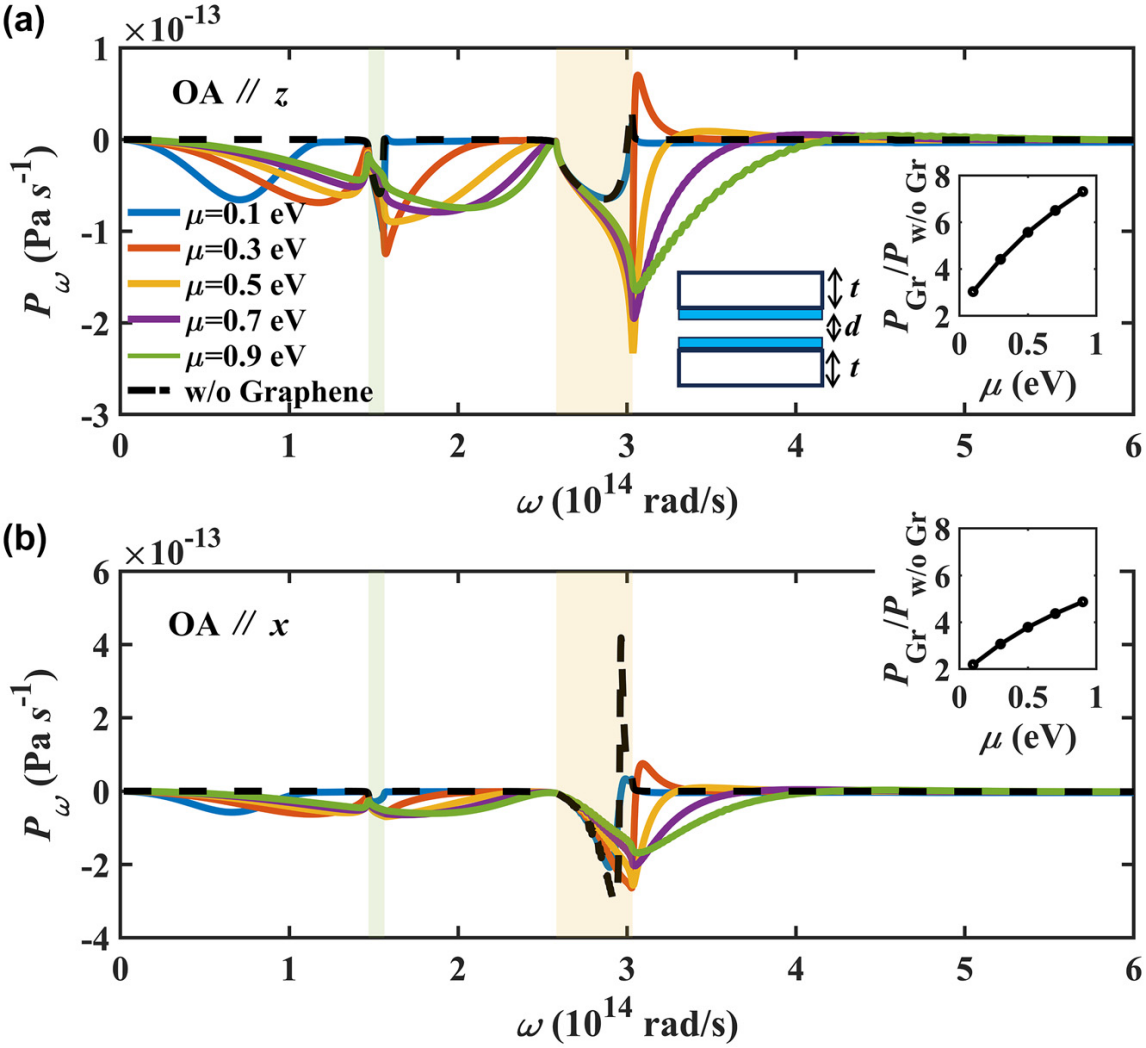
The spectral Casimir force between hBN bulk covered by graphene varying with frequency when OA is along (a) the *x*-axis and (b) the *z*-axis. The insets represent the ratio of Graphene/hBN to bare hBN (w.o Gr) total Casimir force with chemical potential.

The distribution of spectral Casimir forces between hBN bulks covered by graphene with different chemical potentials are shown in Figure 10. It can be clearly seen that the Casimir force of bulk hBN is only promoted in the HBs (shaded areas) due to its hyperbolic dispersion. When covering the graphene, the Casimir force can be greatly enhanced beyond the HBs. Similar phenomena have been observed in the hBN/*α*-MoO_3_ covered with graphene due to the coupling of surface plasmons in graphene and HPs in HMs [41], [50], [58]–[60]. The total Casimir forces exhibit a monotonic increase with the chemical potential of graphene as shown in the inset. When OA is out-of-plane, the attractive Casimir force at ENZ frequencies is greatly enhanced in the type II HB, enhanced or suppressed in the type I HB, depending on the chemical potential of graphene. The repulsive force at ENZ frequency is much suppressed with high chemical potential. When OA is out-of-plane, the attractive Casimir force is enhanced in the type I HB. While the peak-valley pair in the type II HB is suppressed when adding graphene.

## 4 Conclusions

In summary, the Casimir interaction between natural HM hBN is investigated. The spectral Casimir force between bulk hBN, finite-thickness hBN slabs, and bulk hBN covered with graphene are calculated, respectively. The results show that the ENZ mode could cause a peak-valley pair in the spectrum when OA is out-of-plane. The v-HPs could enhance the attractive Casimir force and lead to an attractive-repulsive transition in the type II HB with finite thickness. When OA is in-plane, the v-HPs could excite type I and II HBs within a certain angle determined by asymptote lines. The s-HPs could excite in the type II HB and greatly enhance the Casimir force. The symmetric and antisymmetric modes of s-HPs cause the attractive-repulsive transition in the spectrum. The shape of the Casimir force distribution in wavevector space changes from hyperbolic to closed hyperbolic at the transition point of the peak-valley pair. When covering the graphene on the hBN, the Casimir force can be greatly enhanced beyond the HB due to the coupling of graphene plasmons and HPs in HMs. This work provides new insights into the Casimir interaction in HM and will help the development of MEMS and NEMS.
